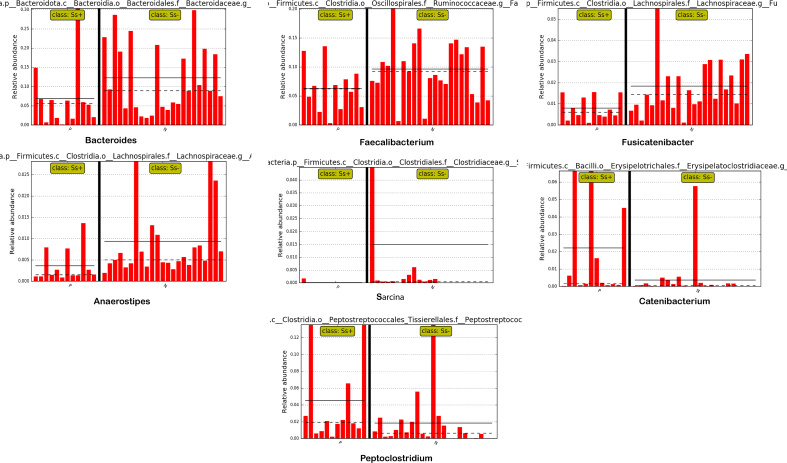# *Strongyloides stercoralis* infection reduces *Fusicatenibacter* and *Anaerostipes* in the gut and increases bacterial amino-acid metabolism in early-stage chronic kidney disease

**DOI:** 10.1016/j.heliyon.2023.e19859

**Published:** 2023-09-06

**Authors:** Na T.D. Tran, Apisit Chaidee, Achirawit Surapinit, Manachai Yingklang, Sitiruk Roytrakul, Sawanya Charoenlappanit, Porntip Pinlaor, Nuttanan Hongsrichan, Hai Nguyen Thi, Sirirat Anutrakulchai, Ubon Cha'on, Somchai Pinlaor

**Affiliations:** a Department of Parasitology, Faculty of Medicine, Khon Kaen University, Khon Kaen, Thailand; b Faculty of Medical Laboratory Science, Danang University of Medical Technology and Pharmacy, Danang, Viet Nam; c Faculty of Public Health, Burapha University, Chonburi, Thailand; d Functional Proteomics Technology Laboratory, National Center for Genetic Engineering and Biotechnology, National Science and Technology Development Agency, Pathum Thani, Thailand; e Centre for Research and Development of Medical Diagnostic Laboratories, Faculty of Associated Medical Sciences, Khon Kaen University, Khon Kaen, Thailand; f Department of Parasitology, Faculty of Basic Medicine, Thai Nguyen University of Medicine and Pharmacy, Thai Nguyen, Viet Nam; g Department of Medicine, Faculty of Medicine, Khon Kaen University, Khon Kaen, Thailand; h Department of Biochemistry, Faculty of Medicine, Khon Kaen University, Khon Kaen, Thailand; i Chronic Kidney Disease Prevention in Northeastern Thailand, Khon Kaen University, Khon Kaen, Thailand

**Keywords:** *Strongyloides stercoralis*, Chronic kidney disease (CKD), Early-stage CKD, high-throughput sequencing, Metaproteomics

## Abstract

Understanding gut bacterial composition and proteome changes in patients with early-stage chronic kidney disease (CKD) could lead to better methods of controlling the disease progression. Here, we investigated the gut microbiome and microbial functions in patients with *S. stercoralis* infection (strongyloidiasis) and early-stage CKD. Thirty-five patients with early stages (1–3) of CKD were placed in two groups matched for population characteristics and biochemical parameters, 12 patients with strongyloidiasis in one group and 23 uninfected patients in the other. From every individual, a sample of their feces was obtained and processed for 16S rRNA sequencing and metaproteomic analysis using tandem liquid chromatography-mass spectrometry (LC-MS/MS). *Strongyloides stercoralis* infection per se did not significantly alter gut microbial diversity. However, certain genera (*Bacteroides*, *Faecalibacterium*, *Fusicatenibacter*, *Sarcina*, and *Anaerostipes*) were significantly more abundant in infection-free CKD patients than in infected individuals. The genera *Peptoclostridium* and *Catenibacterium* were enriched in infected patients. Among the significantly altered genera, *Fusicatenibacter* and *Anaerostipes* were the most correlated with renal parameters. The relative abundance of members of the genus *Fusicatenibacter* was moderately positively correlated with estimated glomerular filtration rate (eGFR) (r = 0.335, p = 0.049) and negatively with serum creatinine (r = −0.35, p = 0.039). *Anaerostipes*, on the other hand, showed a near-significant positive correlation with eGFR (r = 0.296, p = 0.084). Individuals with *S. stercoralis* infection had higher levels of bacterial proteins involved in amino-acid metabolism. Analysis using STITCH predicted that bacterial amino-acid metabolism may also be involved in the production of colon-derived uremic toxin (indole), a toxic substance known to promote CKD. *Strongyloides stercoralis* infection is, therefore, associated with reduced abundance of *Fusicatenibacter* and *Anaerostipes* (two genera possibly beneficial for kidney function) and with increased bacterial amino-acid metabolism in the early-stages of CKD, potentially producing uremic toxin. This study provides useful information for prevention of progression of CKD beyond the early stages.

## 1 Introduction

The impairment of renal structure or function known as chronic kidney disease (CKD) is characterized by an estimated glomerular-filtration rate (eGFR) < 60 mL/min/1.73 m^2^ for three months or longer, regardless of cause [1,2]. CKD is classified into five stages of severity according to the level of eGFR. With rising prevalence, CKD is a global public health issue (more than 800 million people, or 10% of the world's population, are currently affected), high management costs, and poor treatment outcomes [3]. Limiting progression of CKD beyond the early stages (stages 1–3 [4,5]) is an important strategy to slow renal damage and eventual end-stage renal disease (ESRD) or other CKD complications [5]. Patients with severe CKD are more likely to experience negative effects from infections [6] and hence mortality [7]. However, the impact of parasites in early-stage CKD has been little investigated.

Recently, studies have increasingly demonstrated that changes in the gut microbiome can lead to inflammation and CKD in both humans and animals [8,9]. This dysbiosis contributes to an increase in uremic toxins derived from the gut microbiota such as indoxyl sulfate (IS), p-cresyl sulfate (p-CS), and trimethylamine N-oxide (TMAO), which ultimately leads to increased gut permeability though the proliferation of urease-possessing bacteria. In addition, short-chain fatty acids (SCFAs) derived from microbial fermentation of indigestible fiber in the gut, are beneficial in modulating inflammation and oxidative stress. Both uremic toxins and SCFAs produced by bacteria are related to intestinal integrity but in opposing ways [10,11]. When the intestinal barrier is breached, uremic toxins and microbial products are released into the bloodstream. The gut barrier is regulated by fine-tuned interactions between the host immune system and intestinal microbes. Dysbiosis therefore leads to systemic inflammation that is crucial for the progression of CKD [9,10]. Many factors cause gut dysbiosis (and hence possibly progression of CKD), including infections with bacteria, viruses, or parasites.

In Thailand, 11.6 million people (17.5% of the population) currently have CKD [12]. In Khon Kaen, in northeastern Thailand, the prevalence is 26.8% with most individuals having early-stage CKD (stages 1–3) [4]. The high prevalence of CKD patients in this part of Thailand is accompanied by a high prevalence of *Strongyloides stercoralis* infection (15.2%) [4,13,14]. Around 600 million people worldwide are infected by this soil-transmitted helminth [15]. Penetrating through the epidermis, filariform larvae eventually mature in the small intestine as adult females [16]. In chronic infection, infective larvae re-invade the intestinal mucosa or the perineal skin area, leading to an auto-infection cycle. The infections usually result in minor gastrointestinal symptoms and chronic infection with generalized complaints such as gastrointestinal discomfort, bloating, heartburn, irregular diarrhea or constipation, a dry cough, and skin rashes [15]. However, when immunity is weakened by diseases such as CKD or treatment with immunosuppressive drugs, the parasite can quickly multiply, which causes hyperinfection and potentially fatal disseminated strongyloidiasis [17]. *Strongyloides*- or helminth-associated glomerulopathy could be due to direct parasite damage, immunological phenomena, and systemic manifestations [18]. There are several reports of an association between this infection and nephrotic syndrome and minimal-change disease [19,20] as well as alteration of the gut microbiome [14,21,22]. A previous study of CKD patients in Donchang Sub-district, Khon Kaen, Thailand, found that infection with *S. stercoralis* decreased bacterial production of SCFAs and increased the abundance of pathogenic bacteria [14]. These changes could promote the progression of CKD. Hence, understanding the progression of CKD at an early stage, especially when infected with *S. stercoralis*, could lead to better approaches to control the disease. Although otherwise-healthy individuals infected with *S. stercoralis* in Thailand show only minor alterations in their gut microbiome [23], patients with CKD who have immune dysfunction experience alteration in the structure [14] and possibly functional activities of the microbiota, the latter being a possibility we explore in this work. Multi-omics techniques are useful tools to study these changes [24,25]. Future metabolomics research is necessary to determine how *S. stercoralis* infection and CKD are related, which may be mediated by the microbiota.

We hypothesize that alterations in the microbiota caused by *S. stercoralis* infection influence early-stage CKD. Therefore, we studied the relationship between *S. stercoralis* infection and early CKD in terms of gut dysbiosis. Our subjects were from northeastern Thailand. 16S rRNA next-generation sequencing (16S rRNA NGS) was performed on stool samples from patients with early-stage CKD with or without *S. stercoralis* infection to evaluate any association between infection status and renal parameters. Shotgun metaproteomics analysis of fecal samples was also used as a cutting-edge method to investigate the effects of helminth infection on early-stage CKD by gaining insight into the bacterial metaproteome. Findings from the study may assist the hunt for new methods to slow the course of chronic renal disease.

## 2 Materials and methods

### 2.1 Participants and sample collection

Following the principles of the Declaration of Helsinki, the human ethical review committee of Khon Kaen University in Thailand approved this study (HE641434).

Fecal samples were collected during a previous study in Nam Phong and Ubonrat Districts, Khon Kaen Province, northeastern Thailand [13]. After collection, a portion of each fecal sample was rapidly processed to identify *S. stercoralis* infection using the modified agar plate culture (mAPC) technique [13]. Another portion was tested for the presence of parasite eggs and larvae using the formalin-ether concentration technique (FECT) as was previously reported [13]. Most of the remaining sample was frozen (−80 °C) and stored for later analysis.

The current study's inclusion criteria were monoinfection with *S. stercoralis* (absence of any other intestinal parasites) and a diagnosis of CKD stages 1, 2 or 3. The diagnosis was made by a nephrologist for each participant in a previous study [4,14] and was based on clinically proven impairment of renal structure or function, as determined by ultrasonography, and a finding of reduced eGFR. Each individual's CKD (stages 1–3) was graded based on eGFR [1,2]. For those patients with an eGFR >60 ml/min/1.73 m^2^, diagnosis of renal impairment was based on abnormal urine albumin-creatinine ratio (UACR), hematuria, and abnormal renal ultrasonography. These data sets were obtained from CKDNET medical records. The mAPC technique and FECT were used to diagnose intestinal parasite infections from stool samples, as previously reported [13]. Polymerase chain reaction testing, as described below, confirmed that none of the control group members had *S. stercoralis* infection.

Samples from 35 participants were used in this study. Twelve CKD patients with strongyloidiasis (Ss+) were included because they fulfilled the requirements of monoinfection and early-CKD stages (coded as CKD_Ss+). Twenty-three CKD patients without *S. stercoralis* infections (Ss-) made up the control group (coded as CKD_Ss-). Members of the latter group additionally matched the sex, age, and metabolic characteristics of the Ss+ group. (Table 1 and S1 Excel file). Data on differences in population characteristics were analyzed using GraphPad Prism version 8.4 (t-tests and nonparametric tests).

### *2.2* Confirmation of presence or absence of S. stercoralis infection by polymerase chain reaction (PCR)

In order to verify the outcomes of the fecal examination, conventional PCR was performed on DNA extracted from feces. The 18S rRNA gene of *S. stercoralis* (GenBank accession number M84229.1) was amplified using the same primers, amplification protocol as in a prior investigation [26]. PCR products were analyzed using agarose gel electrophoresis and stained to confirm cases of infection.

### 2.3 Fecal DNA extraction, PCR and illumina sequencing

The QIAamp PowerFecal Pro DNA Kit (QIAGEN, Hilden, Germany) was used to extract DNA from all 35 stool samples in accordance with the manufacturer's instructions. Illumina sequencing was as described in a previous study [21]. Briefly, DNA extraction product was checked for quality using a Nanodrop instrument and agarose gel electrophoresis before further steps. The v3-v4 region of the prokaryotic 16S rRNA gene was amplified by PCR using the primers previously published [27] with sample-specific linked barcodes. PCR product from each sample was then pooled, end-repaired, A-tailed, and ligated to Illumina adapters. The library quality was tested before sequencing. The quantified libraries were sequenced on Illumina platforms with a next-generation sequencer (Illumina NovaSeq 6000 System), providing 250bp paired-end raw reads.

### 2.4 Analysis of 16S rRNA NGS sequences

Analysis of raw sequence data was done using QIIME2 version 2021.11 [28]. Raw 16S rRNA reads were first removed by demultiplexing, and samples were then matched to paired-end reads based on their individual barcodes. Quality filtering (Q Score >33) of the raw reads was performed. Quality filtering of the denoised paired-end reads (using DADA2 in QIIME2) was followed by the clustering of the data into operational taxonomic units (OTUs) using a minimum-similarity threshold of 97%. To determine taxonomic identification, Naïve Bayes classifiers using Silva full-length reference (SILVA 138 99% OTUs full-length sequences) were used.

For bacterial diversity analysis, samples were normalized to make comparisons. Chao1, Simpson and Shannon indices all provide comparisons of alpha diversity of OTUs between samples. Non-metric distance scaling (NMDS) was used to display beta diversity. Analysis of the Bray-Curtis dissimilarity distance indices revealed the distribution of samples among groups. How well the ordination describes the observed distances between the samples is indicated by the NMDS plot stress value. Unifrac distance matrices were generated in QIIME2 for comparing biological communities between two groups. Adonis analysis using weighted and unweighted Unifrac matrices was used to examine statistically significant differences in beta diversity. Alpha and beta diversity were displayed using R Studio. Excel was used to create stacked bar graphs showing the relative abundances of the most prominent bacterial phyla and families. Bacterial taxa that differed significantly in abundance between groups were found using a threshold of >2.0 for the linear discriminant analysis (LDA) effect size (LEfSe) score [29].

Comparisons between groups in terms of the most abundant bacterial genera were analyzed using t-tests and plotted as bar graphs using GraphPad Prism v8.4 (GraphPad Software Inc.). Genera that differed significantly in relative abundance between the two groups were evaluated for correlation with eGFR, UACR and CKD stage using nonparametric Spearman correlation in GraphPad Prism version 8.4 (v8.4) (https://www.graphpad.com/). R values < 0 and r values > 0 indicate negative and positive correlations, respectively. R values of 0.01–0.19, 0.2 to 0.29, 0.3 to 0.39, 0.4 to 0.69, ≥0.7 indicate no or negligible correlation, weak correlation, moderate correlation, strong correlation, and very strong correlation, respectively [30]. A p-value <0.05 was interpreted as a statistically significant difference. Data on relative abundance and renal parameters are provided in Excel file S2.

### 2.5 Sample preparation for shotgun proteomics

Total protein from 100 mg feces was isolated using 0.5% sodium dodecyl sulfate and precipitated with two volumes of cold acetone at −20 °C overnight. The protein pellet was collected after centrifugation and protein concentration was determined by Lowry assay using bovine serum albumin as the protein standard [31]. Proteins were digested into tryptic peptides by trypsin for 16 h at 37 °C and akylation with iodoacetamide. The tryptic peptides were then dried and resuspended in 0.1% formic acid for nano-liquid chromatography tandem mass spectrometry (nano LC-MS/MS) analysis.

### 2.6 Liquid chromatography-tandem mass spectrometry (LC/MS-MS)

Samples of tryptic peptide were examined using the Thermo Scientific Ultimate3000 Nano/Capillary LC system (Thermo Scientific, UK) coupled to a hybrid quadrupole Q-ToF Impact II (Bruker Daltonics Ltd; Hamburg, Germany) with a nano captive-spray ion source. Peptide digests were enhanced on a μ-precolumn 300 μm i. d. X 5 mm C18 Pepmap 100, 5 μm, 100 Å (Thermo Scientific, UK) and separated on a 75 μm I.D. x 15 cm column and packed with Acclaim PepMap RSLC C18, 2 μm, 100 Å, nanoViper (Thermo Scientific, UK). The C18 column was surrounded with a column oven set at 60 °C. Solvents A and B were supplied with 0.1% formic acid in water and 0.1% formic acid in 80% acetonitrile, respectively, for the analytical column. Peptides were eluted for 30 min at an unchanged flow rate of 0.30 μl/min using a gradient of 5–55% solvent B. At 1.6 kV, electrospray ionization was accomplished using CaptiveSpray. Nitrogen was used as the drying gas at a rate of around 50 l/h. Collision-induced dissociation (CID) product ion mass spectra were received using nitrogen gas as the collision gas. Over the (*m*/*z*) range of 150–2200, positive ion mode mass spectra (MS) as well as MS/MS spectra were recorded at 2 Hz. According to the *m*/*z* value, the collision energy was adjusted to 10 eV. LC-MS was used to evaluate each sample three times.

### 2.7 Metaproteomic analysis

Individual samples were quantified using MaxQuant 2.1.0.0. Their MS/MS spectra were matched to Uniprot using the Andromeda search engine to identify the proteins of the 10 most abundant bacterial families from the Uniprot database (accessed on December 15, 2022). Using the default parameters of MaxQuant, carbamidomethylation of cysteine was employed as a fixed modification, methionine oxidation and acetylation of the protein's N-terminus as variable modifications, and trypsin as the digestive enzyme. For protein identification and additional data analysis, peptides containing at least seven amino acids were taken into consideration. A 1% false discovery rate (FDR) was specified. A maximum of five changes could be made to a peptide. Following the collection of spectral data on the total proteins expressed by each bacterial family, the peptides with the highest intensity from three injections were found. Some peptides matched a protein from a single bacterial strain, allowing low-level taxonomic classification.

The log2-transformed maximum peptide intensities provided protein expression levels for quantifying protein quantities and analyzing differentially expressed proteins (DEPs) in the ten most-common bacterial families. Multiple t-tests using the two-step linear step-up procedure of Benjamini, Krieger and Yekutieli [32] (FDR = 1%) in GraphPad Prism were applied to compare the numbers of proteins in these bacterial families in the two sample groups. The number of proteins in the two groups is provided in the S3 Excel file. Then, in order to find DEPs, the log2 of the maximum peptide intensity of each sample was statistically analyzed (multiple t-tests) with FDR = 10% in GraphPad Prism v8.4 [33]. Genes encoding DEPs that were more abundant in the CKD_Ss+ group were analyzed for functional annotation and enrichment using the DAVID v2022q3 (https://david.ncifcrf.gov/home.jsp). The summary and classification of biological functions of DEPs in CKD patients with strongyloidiasis was presented in a pie chart. DEPs relating to SCFA metabolism and amino-acid metabolism were further investigated. Uremic toxin-related proteins were displayed in bar graphs using GraphPad Prism version 8.4. The STITCH (version 5) database (http://stitch.embl.de/) was used to predict functional interaction networks among identified proteins related to amino-acid metabolism and uremic toxins and to generate a protein-correlation map [34].

## 3 Results

### 3.1 Study population characteristics

Age, sex, BMI and biological test results were matched between the two CKD groups with and without *S. stercoralis* infection, as shown in Table 1. The percentage of eosinophils in the CKD_Ss+ group was significantly greater (p = 0.007), a common finding in allergic or parasitic diseases. This eosinophilia was also the cause of significantly higher total white blood-cell counts (p = 0.02) in the CKD_Ss+ group. Other parameters did not differ between the two groups. The FECT results indicated a low burden of parasitic infection in the study participants (S1 Excel file).

**Table 1:** Demographic, physical and biochemical characteristics of participants in the study, showing that the two groups were well matched. (*) and (**) indicate p-values <0.05 and < 0.01, respectively. N is number of samples in each group.

Parameters	CKD and Ss- (Mean ± SD)	CKD and Ss+ (Mean ± SD)	P-value
Sex	Male (n = 7) Female (n = 5)	Male (n = 12) Female (n = 11)	>0.99 >0.99
Age	63.61 ± 5.32	65.33 ± 6.44	0.44
BMI	22.31 ± 4.95	22.08 ± 6.08	0.69
ALT (U/L)	23.02 ± 11.04	24.11 ± 16.25	0.90
Serum Creatinine (mg/dL)	0.99 ± 0.27	1.02 ± 0.31	0.60
Glucose (mg/dL)	104.88 ± 26.3	109.05 ± 28.44	0.44
LDL Cholesterol (mg/dL)	143.27 ± 41.58	119.99 ± 42.01	0.13
Uric Acid (mg/dL)	5.65 ± 1.3	6.19 ± 1.17	0.23
Red Blood cell (RBC) (*10^12^/L)	4.68 ± 0.83	4.66 ± 0.52	0.91
Hemoglobin (g/dL)	12.36 ± 1.44	12.49 ± 1.67	0.82
Hematocrit (%)	38.51 ± 4.41	37.88 ± 4.67	0.61
White Blood Cells (*10^9^/L)	6.39 ± 1.25	8.1 ± 2.1	0.02*
MCV fL	83.67 ± 9.96	82.66 ± 14.95	0.57
MCHC g/dL	32.11 ± 1.32	32.93 ± 1.03	0.05
Platelet (%)	266.48 ± 62.36	294.33 ± 86.56	0.34
Neutrophil (%)	51.79 ± 9.59	46.68 ± 16.25	0.33
Lymphocyte (%)	35.8 ± 9.01	35.62 ± 11.6	0.96
Monocyte (%)	5.89 ± 2.02	5.31 ± 1.39	0.33
Eosinophil (%)	5.75 ± 4.83	11.62 ± 6.23	0.01**
Basophil (%)	0.69 ± 0.41	0.78 ± 0.36	0.41
SBP	146.74 ± 23.09	147 ± 19.83	0.59
DBP	86.22 ± 14.75	88 ± 11.66	0.70
eGFR ml/min/1.73 m^2^	74.5 ± 17.03	71.54 ± 19.44	0.66
UACR	54.67 ± 128.3	42.31 ± 52.67	0.75
CKD stage	1.83 ± 0.65	1.92 ± 0.79	0.79

Data are presented as mean ± standard deviation of the mean. Multiple t-tests with FDR 1% were used to calculate P values. Abbreviations: ALT, alanine aminotransferase; BMI, body mass index; LDL, low-density lipoprotein; SBP, blood pressure systolic (mmHg); DBP, blood pressure diastolic (mmHg); MCV, mean corpuscular volume; MCH, mean corpuscular hemoglobin; MCHC, mean corpuscular hemoglobin concentration; U, urine; UACR, urine albumin-to-creatinine ratio.

### *3.2* *Gut diversity between CKD groups with and without* S. stercoralis

The two groups were compared in terms of alpha and beta diversity. The Chao1 (Fig. 1a, p = 0.89), Shannon (Fig. 1b, p = 0.86), and Simpson (Fig. 1c, p = 0.58) indices showed no difference in alpha diversity between the CKD groups with and without strongyloidiasis. NMDS revealed a wide overlap between the two groups (Fig. 1d). The number in each quadrant of the heat map (Fig. 1e, index = 0.72) is the discrepancy coefficient between the two groups. The larger the value of this coefficient (the maximum value is 1.0), the greater the difference in species diversity. However, Adonis did not find a significant difference between the two groups according to either unweighted Unifrac (R2 = 0.037, p = 0.91) or weighted Unifrac (R2 = 0.052, p = 0.089) distances for beta diversity.

**Fig. 1:**
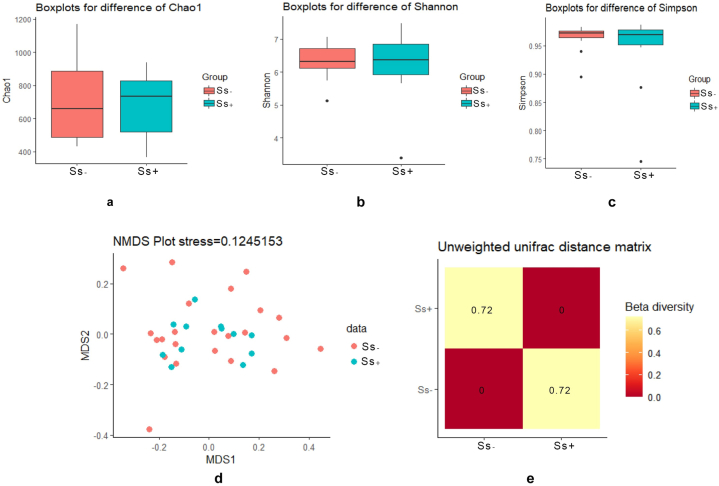
Alpha and beta diversity of CKD groups with or without *S. stercoralis.* A, b, c: Box plots of alpha diversity according to Chao1, Shannon, and Simpson indices. D: NMDS plot demonstrating the distribution of samples between the two sample groups (Ss+, Ss- indicated by red and green dots, respectively). E: Heatmap based on unweighted Unifrac distances between the two groups. The number in each quadrant is the discrepancy coefficient between any two samples.

### 3.3 Most abundant bacterial taxa overall in CKD patients between groups

**Fig. 2:**
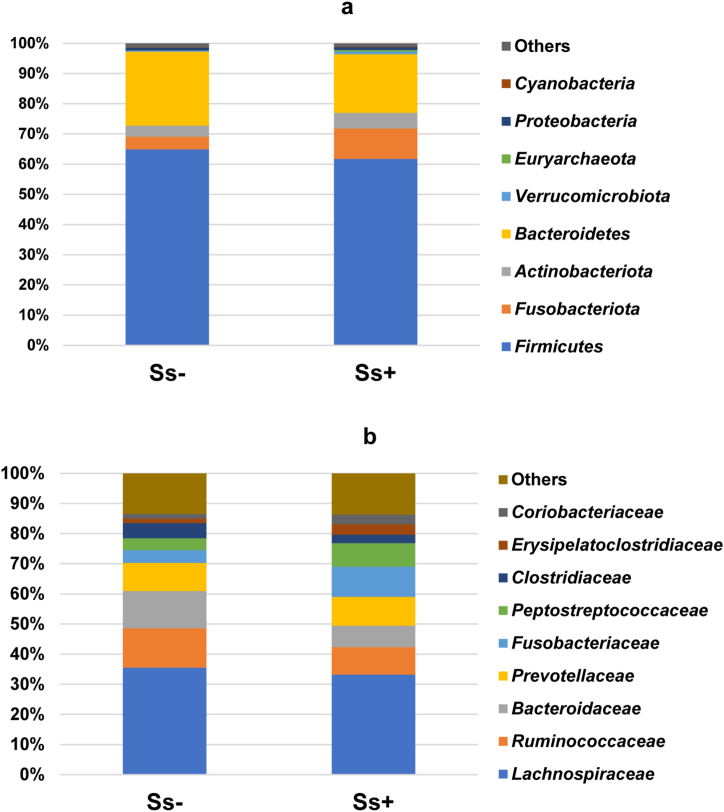
Most abundant bacterial phylum and families. A and b are histograms of relative abundance of bacteria at the phylum and family levels, respectively.

**Fig. 3:**
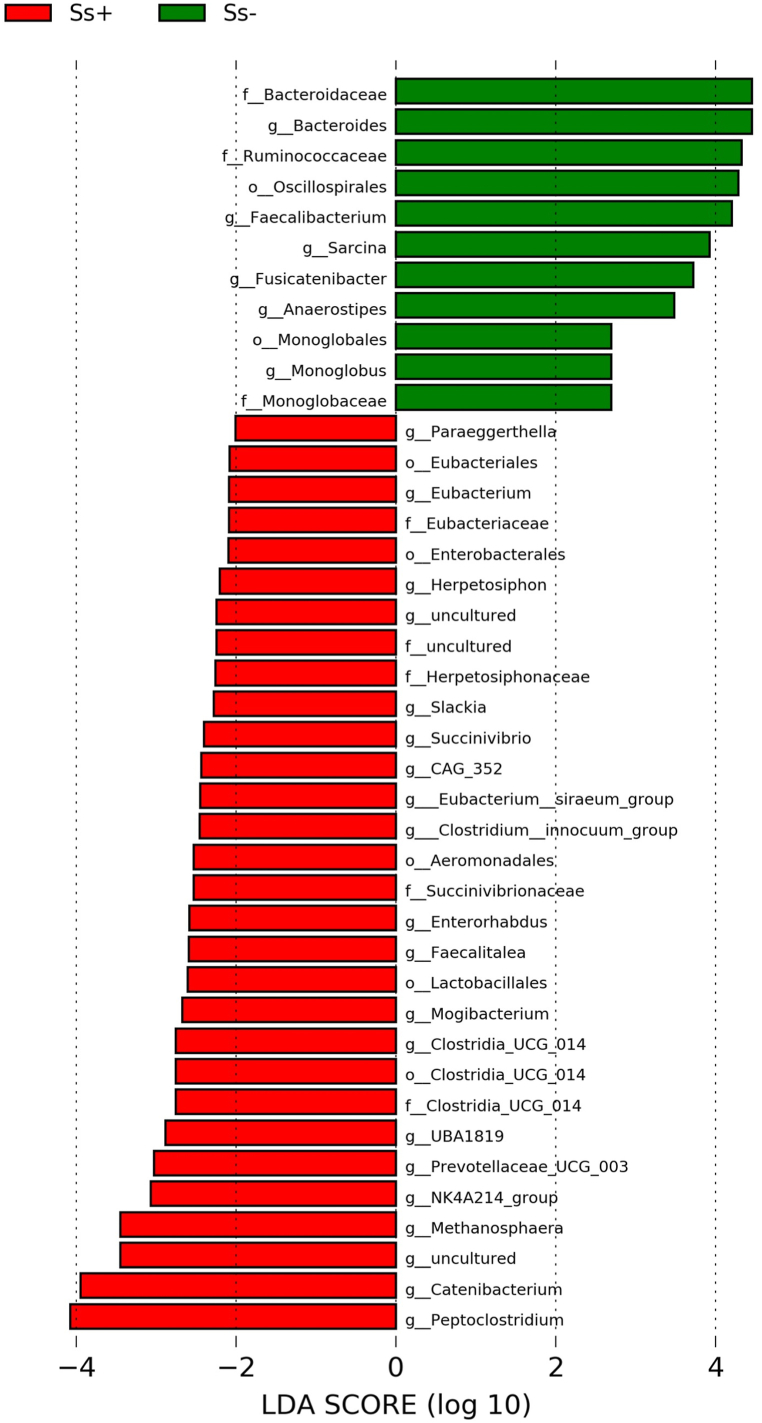
Histograms of linear discriminant analysis (LDA) effect size (LEfSe) comparing stool microbiota of the two groups. Log-level changes in LDA score are displayed on the x axis. Green and red bars indicate taxa found in greater relative abundance in CKD_Ss- and CKD_Ss+ groups, respectively.

The most abundant phyla in the study are shown in Fig. 2a. At the family level, the most abundant families were *Lachnospiraceae, Ruminococcaceae, Bacteroidaceae, Prevotellaceae, Fusobacteriaceae, Peptostreptococcaceae, Clostridiaceae, Erysipelatoclostridiaceae,* and *Coriobacteriaceae* (Fig. 2b). The proportion of *Bacteroidaceae* was significantly lower in the CKD_Ss+ group (7% and 12.4% in the CKD_Ss+ and CKD_Ss- groups, respectively). Similarly, more members of the *Ruminoccaceace* were present in the CKD_Ss- group (13.1% and 9.2% in the Ss- and Ss+ groups, respectively), as revealed by LEfSe analysis (Fig. 3).

### 3.4 Differential representation of genera between groups

**Fig. 4:**
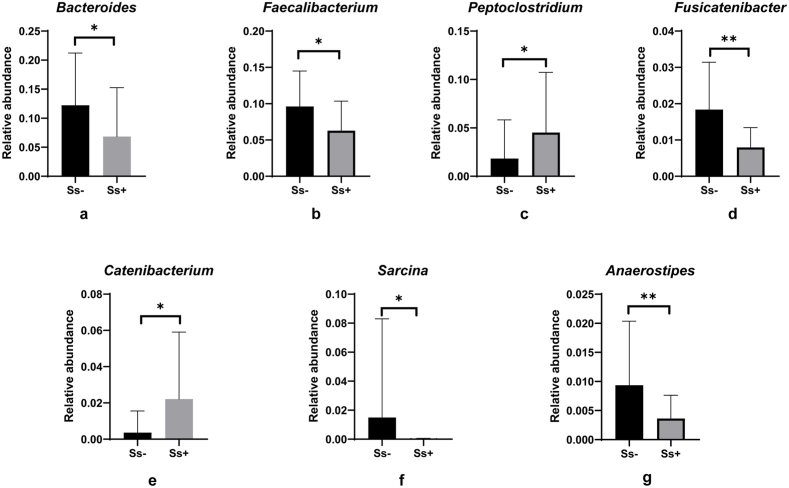
The bacterial genera most altered in abundance between CKD patients with or without *S. stercoralis* infection. The panels compare the relative abundances of *Bacteroides* (a)*, Faecalibacterium* (b)*, Peptoclostridium* (c), *Fusicatenibacter* (d), *Catenibacterium* (e), *Sarcina* (f) and *Aaerostipes* (g) between the two groups. (*) and (**) indicate p-values smaller than 0.05 and 0.01, respectively.

Despite the lack of significant overall differences in alpha and beta diversity, some individual genera differed significantly in abundance between groups. LEfSe indicated many shifts in the abundance of bacterial genera between the two groups (Fig. 3). Among the 35 most abundant genera (S1 Figure), *Bacteroides* (Fig. 4a, p = 0.0485), *Faecalibacterium* (Fig. 4b, p = 0.0413), *Fusicatenibacter* (Fig. 4d, p = 0.0082), *Sarcina* (Fig. 4f, p = 0.0127) and *Anaerostipes* (Fig. 4g, p = 0.0031) were significantly lower in the CKD_Ss + group. On the other hand, *Peptoclostridium* (Fig. 4c, p = 0.0188) and *Catenibacterium* (Fig. 4e, p = 0.0153) were considerately more abundant in the CKD_Ss+ group. *Fusicatenibacter* and *Anaerostipes* were the genera whose abundance changed the most as a result of *S. stercoralis* infection.

### 3.5 Association between changes in the most abundant bacterial genera and renal parameters

Seven bacterial genera (listed above) exhibited significant differences in abundance between the two groups. Data for these genera were evaluated for association with serum creatinine levels, eGFR values, and urine albumin creatinine ratio (UACR) (Table 2). *Fusicatenibacter* showed moderately significant positive and negative correlation with eGFR (r = 0.335; p = 0.049) and serum creatinine levels (r = −0.350; p = 0.039), respectively. The relative abundance of *Fusicatenibacter* showed an almost significant negative relationship with UACR (r = −0.316; p = 0.064). *Anaerostipes* exhibited a near-significant positive association with eGFR (r = 0.296; p = 0.084). Abundance of *Sarcina* was significantly positively correlated with serum creatinine, but significantly negatively correlated with eGFR. However, this genus was generally not very abundant and was found in only a few samples (S2 Figure). The remaining genera showed no correlation with renal parameters (Table 2).

**Table 2:** Correlation between relative abundance (RA) of significant genera with *S. stercoralis* infection and kidney parameters. R-values <0 and r-values >0 indicate negative and positive correlation, respectively. P-value (two-tail) < 0.05 was considered to indicate significant correlation. Symbols (*) and (**) indicate p-values lower than 0.05 and 0.001, respectively.

Genus	RA and serum creatinine	RA and eGFR	RA and UACR
*Bacteroides*	r = −0.06	r = 0.025	r = 0.037
	p = 0.730	p = 0.887	p = 0.831
*Faecalibacterium*	r = 0.084	r = −0.054	r = −0.074
	p = 0.632	p = 0.759	p = 0.672
*Peptoclostridium*	r = 0.146	r = −0.032	r = 0.014
	p = 0.404	p = 0.854	p = 0.936
*Fusicatenibacter*	r = −0.350	r = 0.335	r = −0.316
	p = 0.039*	p = 0.049*	p = 0.064
*Catenibacterium*	r = −0.091	r = −0.034	r = −0.058
	p = 0.602	p = 0.848	p = 0.739
*Sarcina*	r = 0.465	r = −0.420	r = 0.131
	p = 0.005**	p = 0.012*	p = 0.452
*Anaerostipes*	r = −0.258	r = 0.296	r = 0.002
	p = 0.134	p = 0.084	p = 0.911

### 3.6 Identified proteins from the most abundant bacterial families

The total number of proteins from the most abundant bacterial families is shown in Table 3. There was no significant difference in these numbers between CKD patients with and without *S. stercoralis* infection.

**Table 3:** Total numbers of proteins derived from the most abundant bacterial families. Data are presented as mean ± standard deviation of the mean. Multiple t-tests with FDR 1% were used to calculate p-values.

	CKD and Ss-Mean±SD (n = 23)	CKD and Ss+ Mean ± SD (n = 12)	p-value
*Clostridiaceae*	608.5 ± 119.6	590.7 ± 54.7	0.63
*Coriobacteriaceae*	368.9 ± 95	348.6 ± 33.8	0.48
*Fusobacteriaceae*	979.7 ± 242.1	958.6 ± 81.3	0.77
*Lachnospiraceae*	600.8 ± 146.1	574.3 ± 36	0.54
*Peptostreptococcaceae*	271.1 ± 66.7	257.7 ± 23.1	0.50
*Ruminococcaceae*	1086.4 ± 252.5	1033.5 ± 85.8	0.50
*Bacteroidaceae*	1281.4 ± 331.4	1223.1 ± 85.9	0.56
*Erysipelatoclostridiaceae*	641 ± 166.5	633.8 ± 37	0.88
*Prevotellaceae*	2127.4 ± 482.9	2041 ± 210.9	0.56

**Fig. 5:**
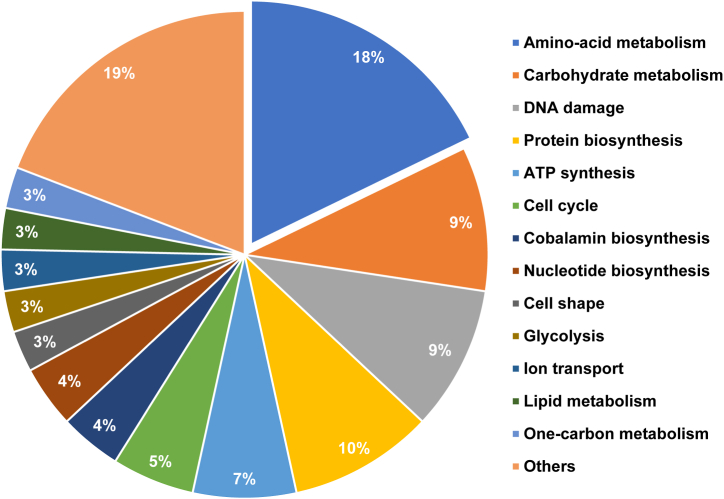
Biological functions of 110 proteins differently expressed at higher levels in CKD patients with *S. stercoralis* infection according to DAVID.

**Fig. 6:**
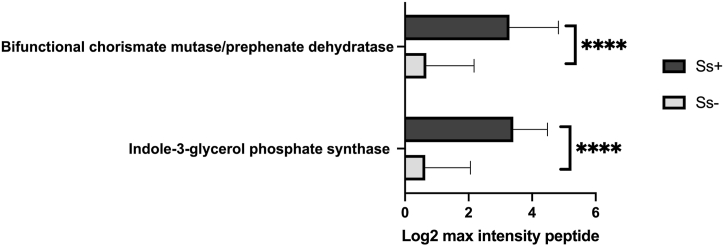
Comparisons between groups of two proteins associated with metabolism of colon-derived uremic toxins with FDR testing. (**** indicates p-value <0.001).

**Fig. 7:**
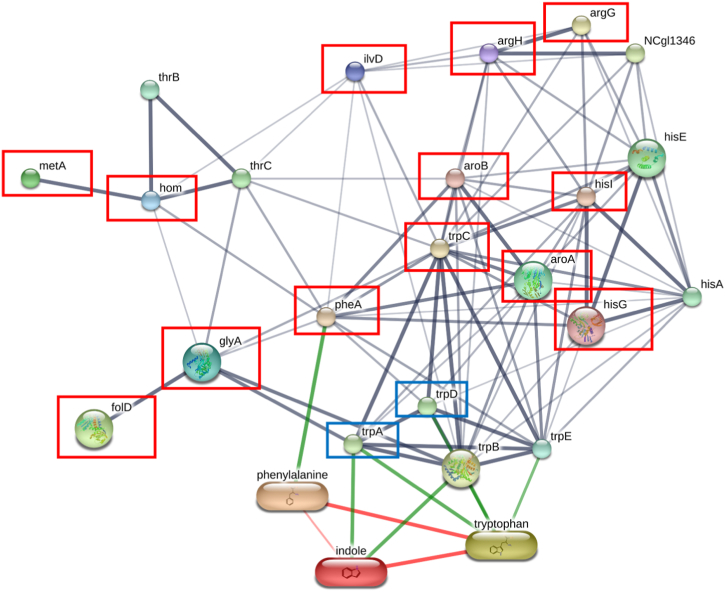
STITCH predictions of proteins associated with amino acid metabolism and indole production. The red boxes enclose names of genes listed in Table 4. The blue boxes enclose names of genes significantly upregulated in CKD_Ss+ (without FDR testing). *Corynebacterium glutamicum* was used as the template taxon to generate the figure.

The DEP analysis revealed 1325 individual proteins (S4 Excel file) that were significantly different between the two groups. Among these, 1192 proteins (S5 Excel file) were elevated in the CKD_Ss+ group, 110 of which were categorized by biological function using the DAVID database (Fig. 5), indicating that amino-acid metabolism was most enriched in the CKD_Ss+ group. Given our hypothesis that the CKD_Ss+ group has increased bacterial metabolites such as indoles, and *p*-cresol that contribute to uremic toxicity, amino-acid metabolism by bacteria was further explored. Interestingly, two proteins involved in the metabolism of colon-derived uremic toxins differed very significantly in intensity between the two groups, as shown in Fig. 6. Twelve proteins associated with amino-acid metabolism (Table 4) were predicted to interact with production of uremic toxins (indole metabolism) according to the STITCH analysis (Fig. 7).

**Table 4:** Genes encoding differentially expressed proteins related to amino-acid metabolism found at significantly higher levels in the CKD_Ss+ group according to DAVID.

ID	Gene Name	Species	p-value
SAMN02910436_00940	Serine hydroxymethyltransferase (glyA)	*Ruminococcaceae bacterium P7*	<0.001
CLK_2057	Argininosuccinate lyase (argH)	*Clostridium botulinum A3 str. Loch Maree*	0.002
CA_C0894	3-dehydroquinate synthase (aroB)	*Clostridium acetobutylicum DSM 1731*	0.004
CKR_0704	3-phosphoshikimate1-carboxyvinyltransferase (aroA)	*Clostridium kluyveri NBRC 12016*	<0.001
B6259_08880	Bifunctional chorismate mutase/prephenate dehydratase (B6259_08880)	*Ruminococcaceae bacterium CPB6*	<0.001
CA_C3160	indole-3-glycerol phosphate synthase TrpC (trpC)	*Clostridium acetobutylicum DSM 1731*	<0.001
SAMN02745687_02279	Dihydroxy-acid dehydratase (SAMN02745687_02279)	*Lachnospiraceae bacterium NK3A20*	<0.001
Cbei_1323	phosphoribosyl-AMP cyclohydrolase (hisI)	*Clostridium beijerinckii*	<0.001
CLJ_B1673	ATP phosphoribosyltransferase (hisG)	*Clostridium botulinum Ba4 str. 657*	<0.001
Cbei_1702	Bifunctional protein FolD (folD)	*Clostridium beijerinckii NCIMB 8052*	<0.001
CLD_3397	Bifunctional protein FolD (folD)	*Clostridium botulinum B1 str. Okra*	<0.001
CLL_A2913	Homoserine *O*-acetyltransferase (metAA)	*Clostridium botulinum B str. Eklund 17B (NRP)*	0.002

## 4 Discussion

In this study, we investigated the gut microbiomes in patients with early-stage CKD, with or without infection with *S. stercoralis*, using 16S rRNA sequence data and metaproteomics, thus providing deep insights into the complex functional interplay between microbes, their hosts and infection with this parasitic nematode.

We found no significant changes in alpha or beta diversity of bacterial taxa as a result of *S. stercoralis* infection in CKD patients. A previous cross-sectional study of patients with CKD (all stages) and *S. stercoralis* infection in Donchang Sub-district showed a reduction in alpha diversity in infected individuals [14]. The present study extends this work by confirming the effects of *S. stercoralis* infection in early-stage CKD. The differences between these studies may be due the fact that we only used samples from patients with early-stage CKD. The severity of CKD correlates with the structure and diversity of the fecal microbial community [35]. The intensity of parasite infection affects the diversity of the gut microbiome [36]. A slight change in gut microbiome diversity in this study could be explained by our observation of a low parasite burden in most cases. Thus, mild infection with *S. stercoralis* in early-stage CKD may not lead to overall significant changes in gut microbiome diversity. A recent study from the same region of Thailand also found no significant differences in gut microbiome structure and diversity between healthy individuals with or without *S. stercoralis* infection [23]. Recently, a longitudinal study found no significant changes in gut diversity before and after treatment of *S. stercoralis* infection in healthy individuals [37].

In patients with *S. stercoralis* infection in early-stage CKD, the abundance of seven bacterial genera changed significantly (compared with uninfected individuals). Several of these genera (*Feacalibacterium, Fusicatenibacter* and *Anaerostipes*), known to produce short-chain fatty acids, were present in lower abundance in the CKD_Ss+ group. Previously, it was shown that serum SCFAs were reduced in patients infected with *S. stercoralis* [21] and in CKD patients [38]. SCFAs are known for their effects not only on the healthy gut microbiome but also on renal function [39,40]. *Faecalibacterium* is also associated with CKD progression [[41], [42], [43]].

*Fusicatenibacter* and *Anaerostipes* were the most depleted bacterial genera in our CKD_Ss+ group, and their changes in abundance correlated with renal parameters. Increasing relative abundance of *Fusicatenibacter* was significantly associated with higher eGFR and lower serum creatinine and UACR values. Members of the genus *Fusicatenibacter* are anaerobic sugar fermenters [44] and are depleted in CKD and hemolysis patients with low SCFA levels [45]. *Fusicatenibacter* is able to protect humans from kidney stones [46,47]. The genus *Anaerostipes* exhibited a nearly significant positive correlation with eGFR. In a previous study in Donchang, CKD patients with strongyloidiasis had lower abundance of the genus *Anaerostipes* compared to CKD patients without *S. stercoralis* infection [14]. In another study, it was found that increased abundance of the genus *Anaerostipes* was associated with higher eGFR [48]. A decrease in *Anaerostipes* abundance was also associated with an increased risk of death in hemodialysis patients [49]. In addition, *Anaerostipes* showed a strong negative correlation with serum indole sulfate, a uremic toxin [50]. Decreased abundances of *Fusicatenibacter* and *Anaerostipes* were associated with decreases in eGFR, albumin and acetic acid in CKD patients on a low-protein diet [51]. Thus, *Fusicatenibacter* and *Anaerostipes* are both beneficial to the kidney, and their relative abundances were reduced in individuals with *S. stercoralis* infection in early-stage CKD compared to individuals without infection. Both bacteria were also significantly decreased in patients with diarrhea [[52], [53], [54]], which is a known symptom of *S. stercoralis* infection. In most cases in this study, *S. stercoralis* infection was detected in loose stools. A placebo-controlled study of chronic diarrhea showed an increase in the abundance of *Anaerostipes* in parallel with improvement in symptoms of chronic diarrhea in a group receiving probiotics [55,56]. The gut microbiota varies greatly by geographic location, but *Fusicatenibacter* and *Anaerostipes* were consistently reduced in renal failure and diarrhea in many regions [14,45,[48], [49], [50], [51], [52], [53], [54], [55], [56]]. These bacteria may play an important role in maintaining gut health as well as the gut-kidney axis. Further studies using several robust techniques are needed to clarify the role of these bacteria in renal failure alone or with chronic diarrhea and to investigate possible synbiotic treatment with these bacteria in renal disease.

Interestingly, we found that *Peptoclostridium* was increased in the CKD_Ss+ group. *Peptoclostridium* is a genus that includes *P.* (*Clostridium*) *difficile*, an important nosocomial pathogen that causes diseases ranging from antibiotic-associated diarrhea to life-threatening pseudomembranous colitis [57,58]. Although *Peptoclostridium* was not correlated with any renal parameters in this study, the genus is significantly more abundant in patients with renal impairment [59,60]. *Peptoclostridum* and *Escherichia-Shigella* were increased in our CKD_Ss+ group (Fig. 3), and both are important triggers of intestinal inflammation [61,62], which may have deleterious effects on renal function.

Metaproteomics is a useful tool for understanding the impact of parasitic infection on other diseases. Our study is the first to perform metaproteomics research in CKD with *S. stercoralis* infection. Some proteins, although present in both groups, were significantly increased in the CKD_Ss+ group and were related to amino-acid metabolism (Table 4). An increase in intestinal amino-acid metabolism has been associated with the progression of CKD in rats [63]. Specifically, the elevated proteins were expected to stimulate indole (tryptophan) production (gene name: indole-3-glycerol phosphate synthase) and phenylalanine production (bifunctional chorismate mutase/prephenate dehydratase). Both are metabolically linked to uremic toxins from the colon [64]. The best-known colon-derived uremic toxins, *p*-cresyl sulfate and indoxyl sulfate, increase in concentration with deterioration of renal function [[64], [65], [66]]. Typtophan and phenylalanine are metabolized by intestinal bacteria to indole and/or *p*-cresol, which are then absorbed from the intestine into the blood. In the liver, these are converted to indoxyl sulfate and *p*-cresyl sulfate, respectively [66,67]. Serum levels of these two metabolites are 50- and 10-fold higher, respectively, in patients with chronic renal failure [68,69]. Microbial phenylalanine and tryptophan metabolism increases in CKD patients [50,69,70]. In STITCH analysis, most of the links between proteins of amino-acid metabolism that were elevated in infection and indole metabolism were identified (Fig. 7). It should be noted that indole in mammals is produced only by bacterial metabolic activity because host cells lack the necessary metabolic capacity. Numerous bacterial species, both Gram-positive and Gram-negative, can generate indole, including *Escherichia coli, Proteus vulgaris, Clostridium* spp., and *Bacteroides* [71]. Tryptophanase, a bacterial enzyme, is primarily responsible for intestinal bacteria converting tryptophan into indole (trpA, Fig. 6). We discovered that tryptophanase, an enzyme from the family *Clostridiaceae*, is more prominent in most CKD patients with *S. stercoralis* infection. In view of this, it is possible that members of *Clostridiaceae* were the major source of indole in the gut of these patients. Further techniques are required to investigate the concentration of uremic-toxin metabolites in CKD patients with *S. stercoralis* infection.

In the bacterial proteomes of patients with early-stage CKD who were infected with *S. stercoralis*, we found no decrease in proteins associated with SCFA production (listed in S4 Excel file). It is possible that the combination of early-stage CKD and mild infection was insufficient to cause such a change. A limitation of this study is that we did not measure metabolic concentration of SCFAs in feces or of uremic toxins in serum or urine. Evaluation after ivermectin treatment could confirm the role of *S. stercoralis* infection in accelerating the progression of CKD beyond the early stages. The study has limitations because of the small number of samples available to us. This was due to the lockdown in Thailand during the COVID-19 pandemic and the stringent sample selection criteria, which focused only on early-stage CKD with monoinfection of *S. stercoralis*. It is obvious that more research is needed to determine how CKD and *S. stercoralis* infection are related.

## 5 Conclusions

Our study of early-stage CKD patients with *S. stercoralis* infection in communities in northeastern Thailand showed that the presence of this infection did not significantly alter overall gut microbial diversity. However, early-stage CKD patients with *S. stercoralis* infection were found to be depleted of *Faecalibacterium, Fusicatenibacter*, and *Anaerostipes*, all known to produce SCFAs. *Fusicatenibacter* and *Anaerostipes* were associated with renal parameters indicative of CKD and were responsible for most the changes in abundance of bacterial composition observed in *S. stercoralis* infection. Metaproteomics was used to discover that early CKD with parasitic infection boosts bacterial amino-acid metabolism in the gut, potentially enhancing production of uremic toxins from the colon. Thus, both 16S rRNA sequencing and metaproteomic analysis of CKD patients with strongyloidiasis revealed a reduction in bacteria that are beneficial for the kidney and increased bacterial amino-acid metabolism in the early stages of CKD. Further study could confirm the adverse effects of *Strongyloides* infection in early-stage CKD patients before and after treatment for the parasites. Moreover, the functions of *Fusicatenibacter* and *Anaerostipes* in renal failure alone or with chronic diarrhea should be explored to find ways to slow down the progression of CKD.

## Funding

This research was supported by the Fundamental Fund of 10.13039/501100004071Khon Kaen University (FF66), which received funding from The National Science Research and Innovation Fund (NSRF). S.A. acknowledges the 10.13039/501100017170Thailand Science Research and Innovation (10.13039/501100017170TSRI), through Program Management Unit for Competitiveness (PMUC), number C10F630030 and CKDNET (grant no. CKDNET2559007) and Chronic Kidney Disease Prevention in the Northeast Thailand research project, 10.13039/501100004071Khon Kaen University, Thailand (CKDNET2019).

## Author contribution statement

Na T. D. Tran: Conceived and designed the experiments; Performed the experiments; Analyzed and interpreted the data; Wrote the paper.Apisit Chaidee: Conceived and designed the experiments; Wrote the paper. Manachai Yingklang: Performed the experiments; Analyzed and interpreted the data; Wrote the paper. Sitiruk Roytrakul: Performed the experiments; Analyzed and interpreted the data; Contributed reagents, materials, analysis tools or data; Wrote the paper. Sawanya Charoenlappanit: Nuttanan Hongsrichan: Achirawit Surapinit: Performed the experiments. Porntip Pinlaor: Ubon Cha'on: Sirirat Anutrakulchai: Contributed reagents, materials, analysis tools or data. Hai Thi Nguyen: Performed the experiments; Wrote the paper. Somchai Pinlaor: Conceived and designed the experiments; Contributed reagents, materials, analysis tools or data; Wrote the paper.

## Data availability statement

Data associated with this study has been deposited at All sequence reads have been deposited at the NCBI Sequence Read Archive (SRA) under project accession number.

PRJNA924536 (https://www.ncbi.nlm.nih.gov/bioproject/PRJNA924536). The MS/MS raw data and analysis files have been deposited in the ProteomeXchange Consortium.

(http://proteomecentral.proteomexchange.org) via the jPOST partner repository (https://jpostdb.org) with the data set identifier JPST002008 and PXD039656 (preview URL for

Reviewers: https://repository.jpostdb.org/preview/51755362763d13c7fc16bb, Access key: 5345).

## Declaration of competing interest

The authors declare that they have no known competing financial interests or personal relationships that could have appeared to influence the work reported in this paper.

## Figures and Tables

**figs1:**
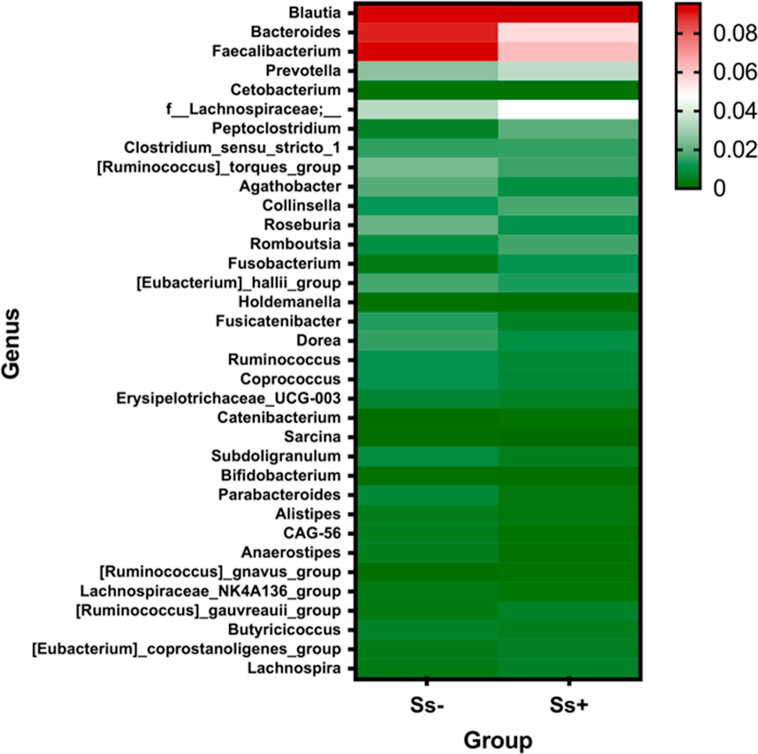


**figs2:**